# Research on Curing Reaction Kinetics and Curing Process of Nitrate Ester Plasticized Polyether (NEPE) Propellants

**DOI:** 10.3390/polym17040464

**Published:** 2025-02-10

**Authors:** Yuheng Wu, Zhiming Guo, Hongjian Yu, Xiaolong Fu

**Affiliations:** 1 Xi’an Modern Chemistry Research Institute, Xi’an 710065, China; wyh915839863@163.com; 2 Department of Civil and Environmental Engineering (DECA), Universitat Politècnica de Catalunya (UPC), Edificio C1, Campus Norte, Jordi Girona 1-3, 08034 Barcelona, Spain; zhiming.guo@upc.edu; 3 School of Mechatronics Engineering, North University of China, Taiyuan 030051, China

**Keywords:** NEPE propellants, curing reaction kinetic, finite element method, curing process

## Abstract

The casting−curing process is a common technology for manufacturing the Nitrate Ester Plasticized Polyether (NEPE) propellants. The curing process involves a coupled thermal-chemical reaction of the adhesive systems of propellant, which influences the curing stage. Using GID 16 software, a propellant grain curing simulation model was conducted. This study employs a model-fitting method based on non-isothermal DSC experiments to analyze the curing reaction kinetics of propellants. Two methods, Kissinger and Ozawa, were used to determine the activation energy of the curing reaction. The reaction activation energy obtained by the Ozawa method was chosen as the simulation parameter Ea = 59.378 based on the fitting coefficients. The simulation comprehensively onsidered flow, temperature, and curing reaction parameters, achieving multi-field coupling of thermal and curing degree fields during the curing process. The macroscopic temperature variations of the pillars were monitored using thermocouples. The experimental results show that the final curing temperature is stable at about 48.2 °C. At about 21,000 s, the overall temperature of the grain converges. The experimental results were compared with the simulation results, revealing minor discrepancies. Experimental and simulation methods were used to verify the changing law of the temperature field inside the propellant grain. Furthermore, these results have significance for improving the casting−curing industrial process of the composite solid propellant.

## 1. Introduction

The NEPE propellant is a critical high-energy solid propellant. It integrates the advantages of both double-base propellants and composite propellants [1]. It offers a combination of high energy and superior mechanical properties. Owing to these exceptional properties, NEPE propellants have seen extensive use in solid rocket motors in recent years [2]. The NEPE propellant is a densely filled solid composite material composed of various components. These include poly(ether-urethane) as the binder, nitric ester serving as the plasticizer, ammonium perchlorate (AP), aluminum particles (Als), and nitramine explosive as solid fillers, along with additional additives. The NEPE propellant belongs to thermosetting materials. Heating curing will form a three-dimensional crosslinking network [3], gradually transformed from the viscoelastic liquid state to a solid state, and its fundamental properties will vary in accordance with the degree of cure [4]. Despite the small proportion of the binder in NEPE solid propellants, the curing network’s characteristics and its reaction kinetics significantly influence the propellant’s process, mechanical, storage, and other properties [5,6]. In order to obtain better propellant properties, the control of the curing reaction rate is very important [7,8]. One method to optimize the curing process is by predicting the temperature and degree of cure variations at different points of the polymer component. This prediction relies on equations and models that describe heat transfer and curing kinetics.

The autocatalytic reaction of solid propellants during the curing process generates a certain amount of heat, and many studies have been carried out on the relationship between the kinetics of the curing reaction and the chemical reaction, time, temperature, and degree of cure [8,9,10]. In a previous study, Fu et al. employed rheometry alongside FTIR spectroscopy to examine the chemical and rheological behaviors of curing reactions [11]. The research demonstrated that the reaction rate of the curing reaction time increases with the increase in the temperature. For the rheological method, Ma et al. study the cure kinetics of the GAP (glycidyl azide polymer) special propellant by rheological isothermal and non-isothermal methods [12]. And Wang et al. [13] investigate the curing kinetics of the GAP/AN/HMX propellant slurries by isothermal rheological measurements. Rheological methods can be used to understand changes in the internal structure of slurry during the curing process. IR is also an important method for assessing the kinetics of propellant curing reactions. Gui et al. [7] investigate the cure kinetics in NEPE propellants by the in–situ microscopic FT-IR method. This study offers a valuable insight into the group changes of the propellant during the curing reaction and derives a kinetic equation for the curing reaction based on the conversion rate. Currently, for the kinetic study of the curing reaction of propellants, the non-isothermal DSC method is usually used for testing due to the rapidity of this method. Zhang et al. employed a non-isothermal DSC method with various scanning rates to analyze the curing kinetics of the HTPB-IPDI curing systems [14]. With an increase in the heating rate, the exothermic peak temperature shifted to higher values, and the shape of the exothermic peak progressively broadened [15,16]. In this study, we have aimed to simulate the curing reaction by obtaining the curing kinetic parameters. There is no in-depth study of the changes at the molecular level during the curing reaction of NEPE propellants, with physical changes in the internal structure of the propellant slurry [17,18,19]. The non-isothermal DSC method is the appropriate method for this study.

In order to predict the temperature and degree of cure changes, many numerical methods have been developed to study the curing process [20,21]. Liu et al. via ABAQUS finite element software studied the residual stress and residual strain in a nitrate ester plasticized polyether propellant grain during the curing and cooling process [22]. In a previous study, the majority of studies have employed a single simulation or experimental method to investigate the curing reaction process of propellants [23]. This paper presents a non-isothermal DSC method to investigate the curing reaction kinetics of NEPE propellants. Additionally, it proposes a combination of experimental and simulation methods to study the curing process of NEPE propellants in an oven.

In this work, the curing reaction process of the NEPE propellant was investigated. Firstly, the curing reaction kinetics of the NEPE propellant was investigated using the non-isothermal DSC method. Through the obtained kinetic parameters of the curing reaction, the thermochemical coupling model of the NEPE propellant was established, and then through the secondary development of GID, the interaction relationship between the degree of curing and temperature was established to simulate the changing law of the propellant degree of curing, temperature, and curing time under different ambient temperatures. With the help of temperature sensors and thermocouples, the distribution of the macroscopic temperature field inside the grain was tested. Finally, the experimental results were compared with the calculated results to summarize the temperature change pattern during the curing process.

## 2. Experiment Setup

### 2.1. Material

**Figure 1:**
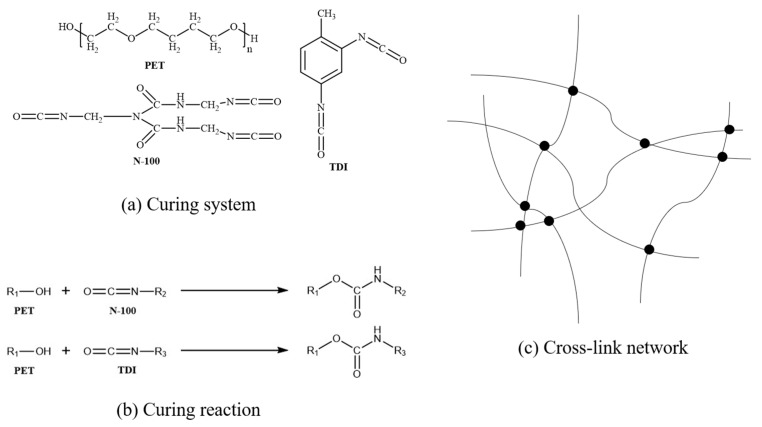
(**a**) Molecular formula of curing system (PET/N-100/TDI). (**b**) Curing reaction between PET and N-100/TDI. (**c**) Cross-link network.

The NEPE propellants consist of tetrahydrofuran copolyether (P(E-CO-T)) as the binder, aluminum (Al) particles with a size range of 6–8 μm as the metal fuel additive, hexamethylene biuret polyisocyanate (N-100) and toluene diisocyanate (TDI) as curing agents, N-butyl-nitroxyethylnitramine (Bu-NENA) as the plasticizer, and ammonium perchlorate (AP) and Octogold (HMX) as substitutes for high-energy explosive additives. The detailed formulation of NEPE propellants is shown in Table 1. Figure 1 illustrates the curing system and reaction.

### 2.2. Sample Preparation

NEPE propellant samples were prepared through a slurry casting process. The initial stage of the procedure involved drying the materials at 50 °C for 10 h. Bu-NENA was dried under reduced pressure. Subsequently, Al powder, AP, HMX, and the curing agent were added to the binder and plasticizer mixture, and the resulting slurry was mixed in a planetary kneader for 1 h. Three 10 mL samples of the homogenized slurry were collected for isothermal rheological testing. The remaining slurry was poured into grain molds for curing experiments.

### 2.3. Curing Experiment

**Figure 2:**
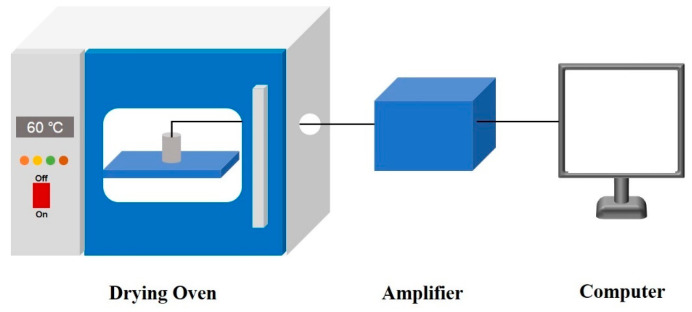
Curing experiment and temperature acquisition device.

The curing experiments were conducted by placing the mixed slurry into a specially designed oven, equipped with a side hub hole for measuring temperature field variations of the grain, at a constant temperature of 60 °C, as shown in Figure 2. A T-type thermocouple was employed for real-time monitoring of the temperature at different locations of the specimen during the curing experiments. T-type thermocouples were utilized for real-time temperature monitoring at various positions of the specimen. The thermoelectric potential signal at the measuring end of the thermocouple was amplified by an amplifier, while an integrated temperature sensor was used to detect the temperature at the reference end.

### 2.4. Non-Isothermal DSC Method

Six milligrams of the stirred propellant slurry was removed from the center and transferred to an aluminum crucible. The temperature was then increased from room temperature to 300 °C under a nitrogen atmosphere. The employed heating rates were 5 °C/min, 10 °C/min, 15 °C/min, and 20 °C/min, aiming to observe the changes in the DSC heat flux at different rates.

## 3. Theoretical Models

### 3.1. Thermo-Chemical Model

The thermo-chemical model consists of heat conduction and curing reaction kinetics. The temperature distribution of the propellant is influenced by the external curing temperature profile and the heat released during the curing reaction, making it a non-linear heat transfer problem with an internal heat source. The thermal analysis is controlled by the balance of energy equation [24]. This governing equation can be stated as follows:

(1) $$\frac{dH}{dt}=-\nabla \cdot q$$

where $\frac{dH}{dt}$ is the enthalpy rate per unit of volume and q is the heat flux.

The enthalpy H (T,α) is the state variable defined as a function of the temperature T and the curing degree α. Therefore, the enthalpy rate in Equation (1) can be written as follows:

(2) $$\frac{dH}{dT}(T,\alpha )=C\frac{dT}{dt}+A\frac{d\alpha }{dt}$$

where C=∂H/∂T is the heat capacity and A=∂H/∂α is the heat provided by the exothermic curing reaction.

The heat capacity of the material is defined as C = ρ⋅c, the product of the material density ρ, and the specific heat c.

The heat flux per unit of surface q is computed as a function of the temperature gradient through the Fourier’s law as follows:

(3) q=−k∇T

where k is the thermal conductivity.

The solution to the thermal problem involves applying the weak form of the energy equation. This requires integrating Equation (1) over the open, bounded volume V, enclosed by smooth boundaries $S=S_{T}\cup S_{q}$ where the corresponding boundary conditions are defined in terms of either the prescribed temperature (T = $\bar{T}$) on $S_{T}$ or prescribed heat flux through the surface Sq with external normal n. The appropriate initial conditions for the transient thermal problem are defined by the initial temperature field: T(t = 0) = $T_{0}$.

The resulting weak integral form of the energy balance equation used for the heat transfer analysis can be written as follows:

(4) $$\int_{V}\left[\left(C\frac{dT}{dt}+A\frac{d\alpha }{dt}\right)\delta T\right]dV+\int_{V}\left[k\nabla T\cdot \nabla (\delta T)\right]dV=W_{ext}(T)$$

where δT is the variation of the temperature field compatible with the Dirichlet’s boundary conditions.$W_{ext}(T)$ represents the external work due to the thermal loads, as follows:

(5) $$W_{ext}(T)=-\int_{S}\left[\left(\bar{q}+q_{cond}+q_{conv}+q_{rad}\right)\delta T\right]dS$$

In Equation (5), $\bar{q}$ represents the prescribed heat flux (Neumann’s condition) while $q_{conv}$ and $q_{rad}$ are the heat fluxes by convection and by radiation, responsible for the heat loss with the environment through the external surfaces of the body.

The effects of the heat convection flux $q_{conv}$ can be considered using Newton’s law (Robin’s condition) in the form [3,4]

(6) $$q_{conv}=h_{conv}(T-T_{env})$$

where $h_{conv}$ is the (temperature-dependent) Heat Transfer Coefficient (HTC) by convection and $T_{env}$ is the environment temperature.

The radiation heat flux $q_{rad}$ can be computed using Stefan–Boltzmann’s law as a function of the propellant surface temperature T and the ambient temperature as follows:

(7) $$q_{rad}=\varepsilon_{rad}\sigma_{0}(T^{4}-T_{env}^{4})$$

where $\sigma_{0}$ is the Stefan–Boltzmann constant and $\varepsilon_{rad}$ is the emissivity parameter.

Finally, the heat flux due to the heat conduction process between the body and the mold surfaces $q_{cond}$ can be taken into account using Newton’s law [3] as follows:

(8) $$q_{cond}=h_{cond}(T-T_{propellant})$$

where $h_{cond}$ is the HTC by conduction between the body and the propellant surfaces in contact, with $T_{propellant}$ being the propellant temperature. The HTC by conduction is defined as the inverse of the corresponding thermal resistivity and it depends on different parameters at the contact interface, such as the contact pressure and the surface’s roughness, among others.

Hence, the discrete form of the balance of energy equation can be written as follows:

(9) $$\sum_{e=1}^{ne}\int_{V(e)}N(e)\left[\rho c\left(\frac{T_{\left(e\right)}^{n+1}-T_{\left(e\right)}^{n}}{\Delta t}\right)-A\left(\frac{\alpha_{\left(e\right)}^{n+1}-\alpha_{\left(e\right)}^{n}}{\Delta t}\right)\right]dV+\sum_{e=1}^{ne}\int_{V(e)}B(e)(k\nabla T_{\left(e\right)}^{n+1})dV=\sum_{e=1}^{ne}W_{\left(e\right)}^{ext}(T)$$

where B(e)=∇N(e) and $\sum_{e=1}^{ne}x$ stands for the assembling procedure.

The curing reaction of an NEPE curing system is typically described using a curing kinetic equation with an Arrhenius-type temperature dependence. The non-isothermal DSC kinetic analysis of the exothermic reaction involves recording the exothermic peak temperature of the reaction at varied heating rates. In the study, the exothermic peak temperature at four different heating rates was recorded to determine Arrhenius activation energy, the pre-exponential factor, and the reaction order through Kissinger and Ozawa methods.

The curing kinetic equation [25] for non-homogeneous systems can be expressed as follows:

(10) $$\frac{d\alpha }{dt}=k(T)f(\alpha )$$

(11) $$k(T)=A\exp\left(-\frac{E_{a}}{RT}\right)$$

E_a_ reflects the difficulty of the curing reaction in the slurries. A larger Ea indicates that the curing reaction is more challenging to achieve. The apparent activation energy of the curing reaction at different curing degrees can be obtained by fitting the curing rate–temperature curves of the slurries using Equation (13).

(12) $$\ln\left(\frac{d\alpha }{dt}\right)=\ln(Af(\alpha ))-\frac{E_{a}}{RT}$$

The curing reaction mechanism of thermosetting resins is commonly described by the autocatalytic model and the n-order reaction model. The experimental data were fitted to both models, and the most suitable model was then selected. For the thermosetting curing reaction, the reaction mechanism is divided into an n-level reaction model and autocatalytic reaction model, and the curing kinetic equation can be constructed according to the shape of its $\frac{d\alpha }{dt}$  − α curve to determine its mechanism function model.

For the n-order reaction model

(13) $$\frac{d\alpha }{dt}=A\exp\left(-\frac{E_{a}}{RT}\right){\left(1-\alpha \right)}^{n}$$

(14) $$\ln\left[\frac{d\alpha }{dt}\exp\left(\frac{E_{a}}{RT}\right)\right]=\ln A+n\ln(1-\alpha )$$

For the autocatalytic model

(15) $$\frac{d\alpha }{dt}=A\exp(-\frac{E_{a}}{RT})\alpha^{m}{\left(1-\alpha \right)}^{n}$$

(16) $$\ln\left[\frac{d\alpha }{dt}\exp(\frac{E_{a}}{RT})\right]=\ln A+n\ln[\alpha^{p}(1-\alpha )]$$

(17) $$p=\frac{m}{n}=\frac{\alpha_{\max}}{1-\alpha_{\max}}$$

where m and n are the reaction orders (1). The relationship of $\ln\left[\frac{d\alpha }{dt}\exp\left(\frac{E_{a}}{RT}\right)\right]$ and ln(1−α) is fitted by Equation (15). The relationship of $\ln\left[\frac{d\alpha }{dt}\exp\left(\frac{E_{a}}{RT}\right)\right]$ and $\ln[\alpha^{p}(1-\alpha )]$ is fitted by Equation (17).

### 3.2. Finite Element Modeling

**Figure 3:**
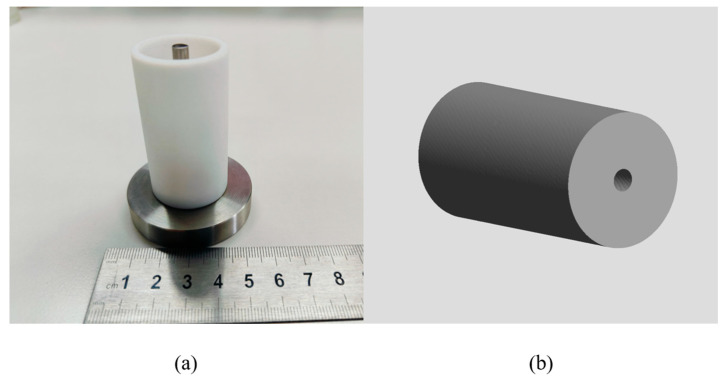
Curing model of NEPE propellant. (**a**) Experiment model. (**b**) Simulation model.

A 3D propellant model was chosen for this structure in order to predict the temperature and curing degree. We modeled the propellant pillars 1:1 in the GID preprocessing module based on the propellant curing model used for the experiments. The NEPE propellant was modeled with 69,432 nodes and 381,742 elements, as shown in Figure 3. And the type of mesh is tetrahedral mesh. After a preliminary study, the number and type of mesh had little effect on the results of the simulation.

Based on the curing process of the NEPE propellant grain, the calculation conditions were defined as follows: for Step 1, the curing temperature T = 50 °C was defined as the initial temperature field. The material parameters of the NEPE propellant are presented in Table 2.

## 4. Results and Discussion

### 4.1. Curing Reaction Kinetics Based on Non-Theermal DSC Method

The primary methods for determining the activation energy of a curing reaction are the Kissinger method and the Ozawa method. The key difference between these methods lies in whether the type of reaction is predetermined. The Kissinger method assumes that the activation energy remains constant throughout the reaction, independent of the degree of cure, and defaults to an n-stage model for the reaction type. In contrast, the Ozawa method applies to a wide range of reaction types and does not require prior determination of the curing reaction type. A comparison of these two calculation methods is presented to select the one most suitable for determining the kinetic activation of the curing kinetics in this material replacement system.

The Kissinger equation can be expressed as follows:

(18) $$\ln\frac{\beta }{T_{p}^{2}}=\ln\frac{AR}{E_{a}}-\frac{E_{a}}{RT_{P}}$$

where β is the heating rate, $T_{P}$ is the peak temperature, A is the pre-exponential factor, E_a_ is the reaction activation energy, and R is the molecular gas constant.

**Figure 4:**
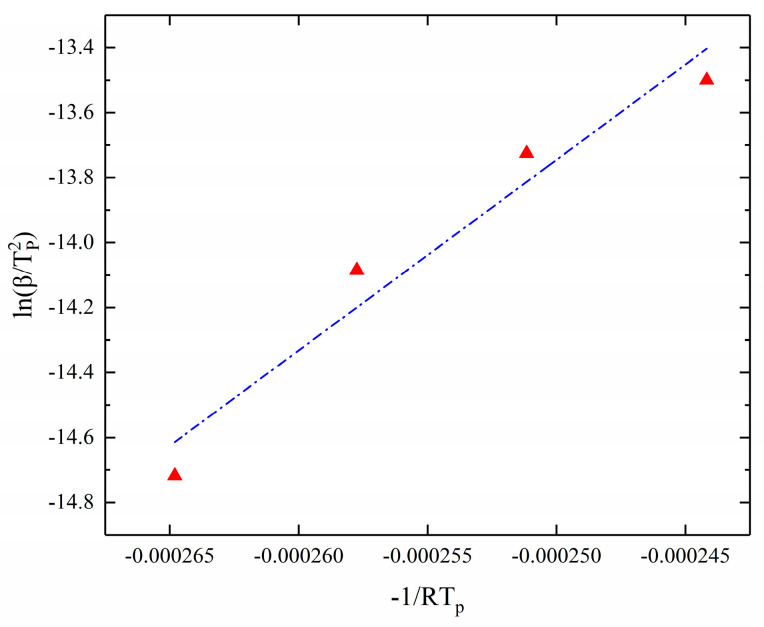
Kissinger method for solving kinetic parameters (−1/RT_p_-$\ln\beta /T_{p}^{2}$ fitting result).

So, the kinetic parameters of the reaction were calculated by the Kissinger method by linear fitting with $\frac{1}{T_{P}}$ as the horizontal coordinate and $\ln\frac{\beta }{T_{p}^{2}}$ as the vertical coordinate. The results of the fitting are shown in Figure 4. The activation energy of the curing process is obtained from the slope of the fitted straight line. In addition, an estimate of A can be obtained from the value.

The Ozawa equation can be expressed as follows:

(19) $$\log\beta =\log\frac{AE_{a}}{Rf(\alpha )}-2.315-0.4567\frac{E_{a}}{RT}$$

where f(α) refers to the mechanism functions for non-homogeneous phase systems.

**Figure 5:**
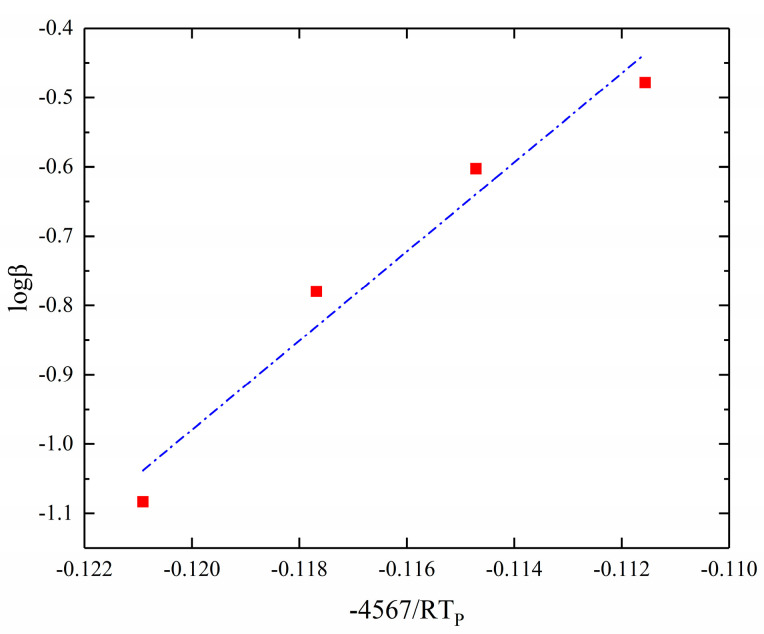
Ozawa method for solving kinetic parameters. (−4567/RT_p_-logβ fitting result).

According to Equation (19), the Ozawa method is used to solve the activation energy of the curing reaction by a linear fit with −4567/RT_p_ as the horizontal coordinate and logβ as the vertical coordinate. The results of the fitting are shown in Figure 5.

By averaging the curing degrees corresponding to the maximum values of the different heating rate functions, from the data in Table 3, the following kinetic equations for the curing reaction process are obtained:

(20) $$\frac{d\alpha }{dt}=21542\exp(-\frac{59.378}{RT})\alpha^{0.58}{\left(1-\alpha \right)}^{1.21}$$

### 4.2. Temperature and Curing Degree During Curing Process

**Figure 6:**
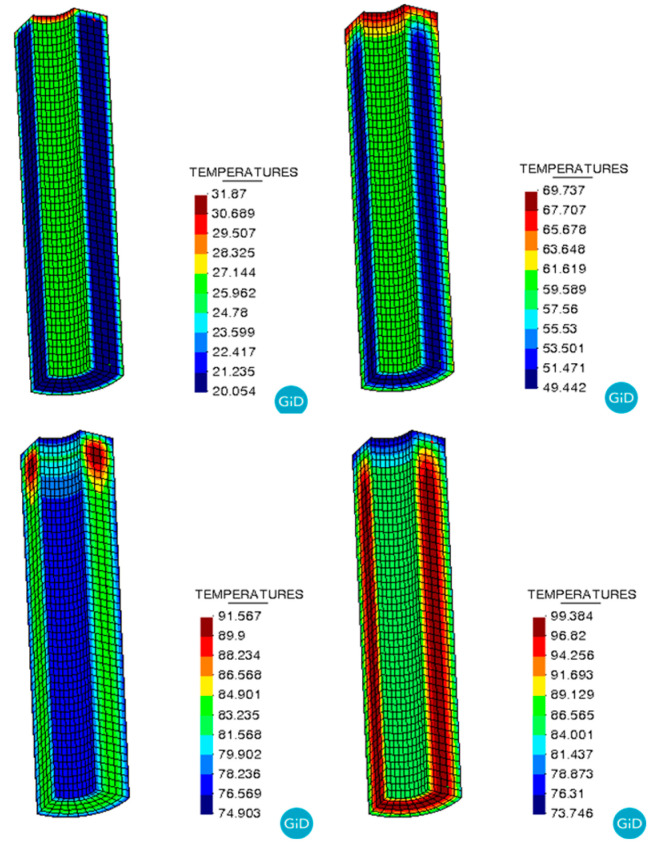
Temperature evolution during the curing process.

Figure 6 presents the temperature of the NEPE propellant grain cured. It is observed that during the curing process, the temperature at the center of the propellant grain is higher than at the periphery. Towards the end of the curing process, the temperature within the grain becomes more uniform.

**Figure 7:**
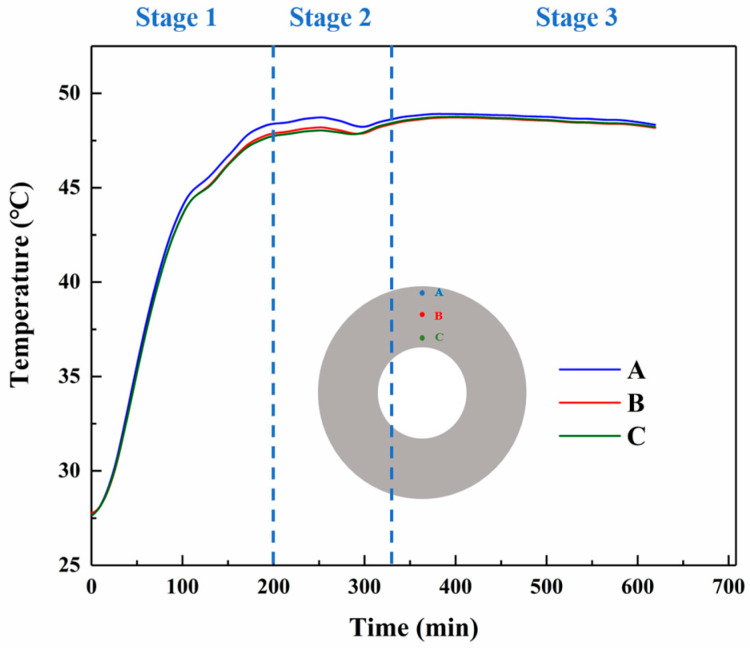
Experimentally tested curves of curing temperature versus time at different nodes.

In the curing experimental setup we built, the temperatures at different locations of the propellant pillars were measured using T-type thermocouples, and the results were in good agreement with the simulation data. Figure 7 shows that the temperature of the outer part of the pillars is higher than that of the inner part, and the final temperature of the pillars tends to converge to the ambient temperature. At the first stage, the curing of propellant samples has a rapid rate of temperature rise. This is mainly due to the heat conduction of the temperature in the oven and the exothermic propellant curing reaction. And the NEPE propellant has better heat transfer properties. In the first stage, the temperature rises to about 47.5 °C at about 200 min. At the second stage, The temperature inside the grain undergoes a slow increase to a maximum and then a slight decrease. This phenomenon can be attributed to the exothermic nature of the curing reaction. The rapid release of heat during the reaction, combined with a lower exothermic rate compared to the ambient heat conduction rate, results in a slight decrease in temperature after the completion of the cross-linking reaction. At the last stage, due to the stabilization of the ambient temperature, the internal temperature of the grain tends to the ambient temperature.

**Figure 8:**
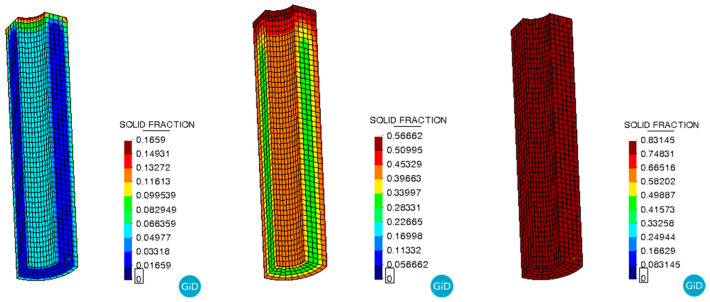
Curing degree during the curing process.

**Figure 9:**
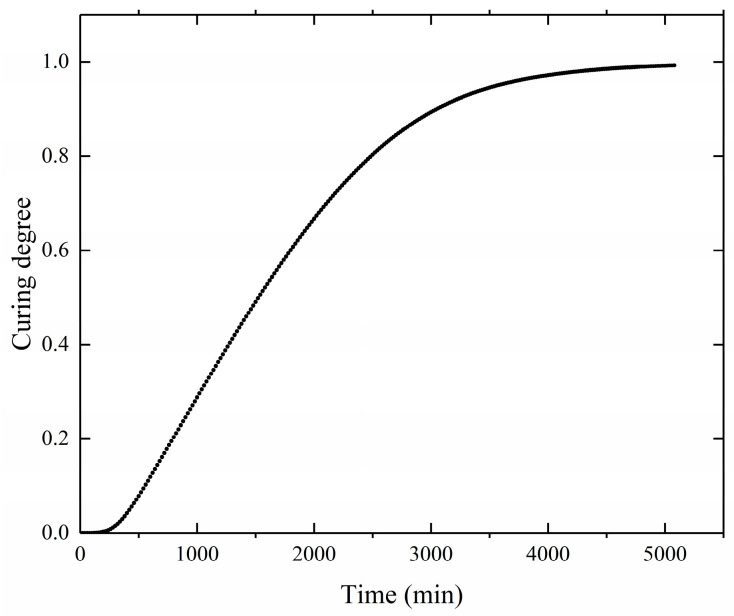
Curing degree curve during the curing process.

The curing degree distribution shows the same trend as shown in Figure 8. As the temperature field distribution, with the center of the propellant grain having a higher degree of curing than the periphery during the curing process, and the degree of curing inside the grain becoming uniform at the end of the curing process. As shown in Figure 9, the curves represent the curing degree versus time curves at location A of the propellant grain monitoring point. After the curing time exceeded 4000 min, the growth of the curing degree began to slow down and gradually tended to stabilize at complete curing. This indicates that most of the reactive components participated in the reaction. The final cure is completed at approximately 5000 min, which is essentially the same as the actual propellant curing process time.

## 5. Conclusions

(1) The curing process of the NEPE propellant system was investigated through non-isothermal DSC measurements using four different heating rates. In this study, the degree of curing was characterized by the exothermic reaction rate, and the kinetic parameters were determined using the Kissinger and Ozawa method. 

(2) During the curing process at 50 °C, a temperature gradient forms within the NEPE propellant grain. The maximum temperature difference is approximately 5 °C, with the highest temperature located at the center of the propellant grain. At the end of curing, the temperature within the grain becomes more uniform. The degree of curing in the NEPE propellant grain follows the same trend as the temperature.

## Figures and Tables

**Table 1:** The materials.

Material	P(E-CO-T)	Bu-NENA	Al	HMX	AP	Others
Content/%	5~10	10~15	10~20	10~15	40~50	1~2

**Table 2:** Material parameters.

Parameter	Value	Unit
Density, ρ	1888	$kg\cdot m^{-3}$
Specific heat, c	1256	$j\cdot {\left(kg\cdot K\right)}^{-1}$
Heat conductivity, s	0.5	$W\cdot {\left(m\cdot k\right)}^{-1}$
Exothermal heat generation, L	10,000	j/kg

**Table 3:** Different methods for solving kinetic parameters.

	Ea/(kJ/mol)	R^2^
Kissinger	59.378	0.9538
Ozawa	61.352	0.9641

## Data Availability

The data presented in this study are available on request from the corresponding author.
